# Supplementary material for: Comparative multiomics analysis of cell physiological state after culture in a basket bioreactor
Source: Sci Rep. 2022 Nov 23;12:20161. doi: 10.1038/s41598-022-24687-4 (PMC9686226; doi:10.1038/s41598-022-24687-4)
Supplement: Supplementary file 1 — Supplementary Information 1. [file 41598_2022_24687_MOESM1_ESM.zip › raw data/Transcriptomic raw data/1.QC/3.Filter/TC1_1_Filter.pdf]

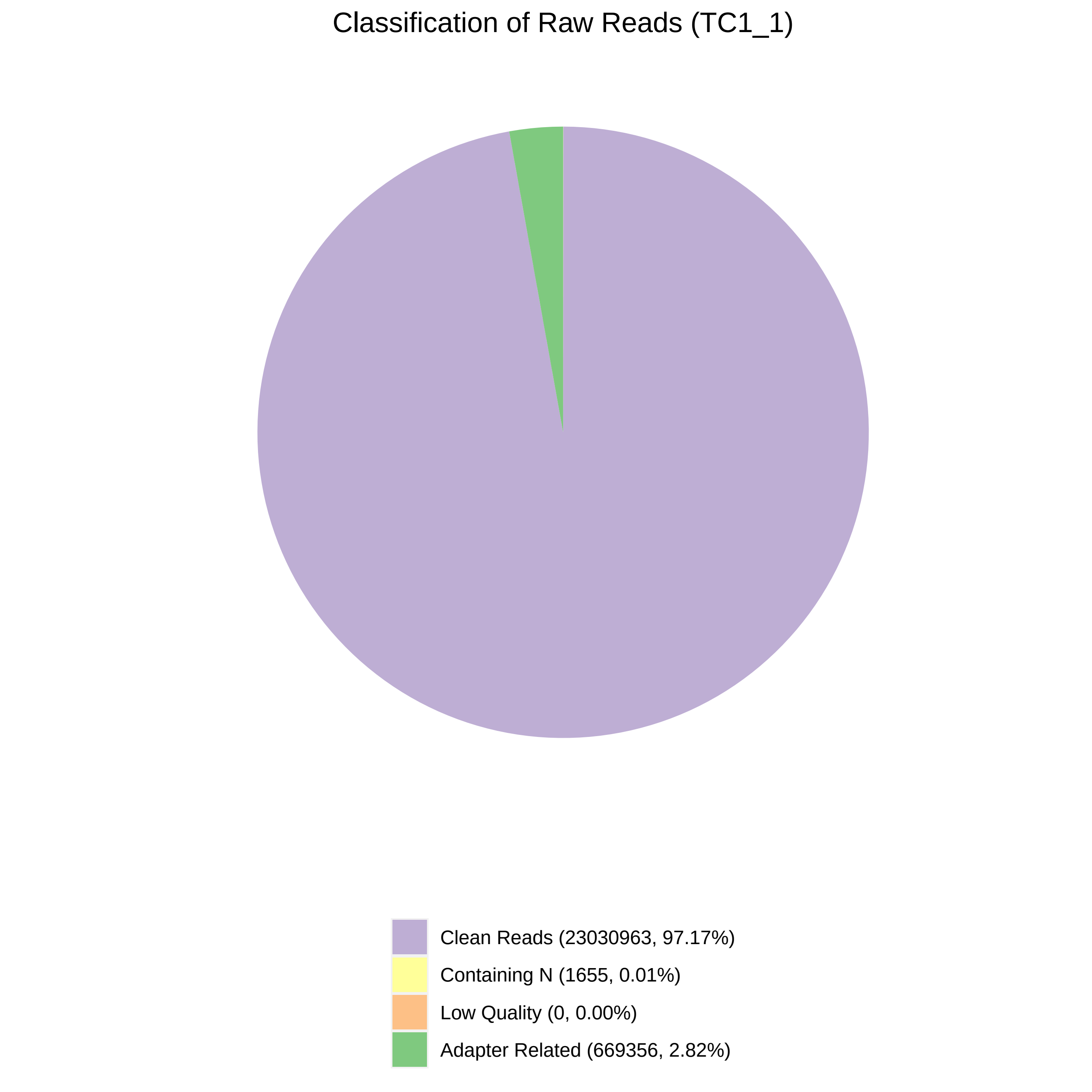
Classification of Raw Reads (TC1_1)
Clean Reads (23030963, 97.17%)
Containing N (1655, 0.01%)
Low Quality (0, 0.00%)
Adapter Related (669356, 2.82%)
